# Assessment of Heavy Metal Pollution in the Sediments of the River Pra and Its Tributaries

**DOI:** 10.1007/s11270-018-3899-6

**Published:** 2018-08-06

**Authors:** Albert Ebo Duncan, Nanne de Vries, Kwabena Biritwum Nyarko

**Affiliations:** 1 Department of Health Promotion, Faculty of Health Medicine and Life Sciences, Maastricht University, B1, 120 Maastricht, Netherlands; 2 Department of Civil Engineering, Kwame Nkrumah University of Science and Technology, Kumasi, Ghana

**Keywords:** Pollution, Heavy metal, Sediments, Illegal mining, Pra River, Ghana

## Abstract

An investigative study was conducted to determine the heavy metal pollution in the sediment in the Pra Basin of Ghana from 27 sampling points during the dry and wet seasons using the geo-accumulation index (Igeo), enrichment factor (EF), and pollution load index (PLI). Sediments were acid digested and analyzed for the following selected metals: arsenic (As), lead (Pb), cadmium (Cd), zinc (Zn), manganese (Mn), total chromium (Cr), nickel (Ni), and iron (Fe) using the dual atomizer and hydride generator atomic absorption spectrophotometer (model ASC-7000 No A309654, Shimadzu, Japan). The metal concentrations (mg kg^−1^) in the sediments were as follows: As (0.175) < Cd (3.206) < Ni (79.927) < Zn (118.323) < Cr (216.708) < Mn (234.742) < Pb (335.381) < Fe (1354.513) in the dry season and As (0.002) < Cd (7.279) < Ni (72.663) < Zn (35.622) < Pb (135.863) < Cr (167.604) < Mn (183.904) < Fe (1138.551) for the wet season. The EF which is an indication of whether metal concentrations are due to anthropogenic activities shows enrichment at all site for the metals Cr, Pb, and Cd in the wet seasons. However, only 4 out of the 27 sites showed Ni enrichment in the wet season. Contrary to the wet season, only Pb and Cr recorded enrichment at all sites during the dry season. Fifteen out of the 27 sites recorded Cd enrichment and 24 out of the 27 sites recorded Ni enriched during the dry season. None of the sites were enriched with Fe, As, Zn, and Mn in either the dry or wet seasons. For both dry and wet seasons, the pollution load index for all the sites except one was at the background levels which is a sign of non-deterioration of the sites studied. In the wet season, the calculated Igeo reveals that the study area is not contaminated with respect to As, Zn, Fe, and Mn; uncontaminated to moderately contaminated with Cd; moderately contaminated with Cr; uncontaminated to moderately to heavily contaminated with Ni; and moderately to heavily contaminated with Pb. The dry season Igeo results reveal non-contamination of the study area with respect to As, Fe, and Mn; uncontaminated to moderately contaminated with Zn; moderately contaminated with Cr; uncontaminated to heavily contaminated with Cd; uncontaminated to extremely contaminated with Ni; and moderately to extremely contaminated with Pb. The high levels of Cd, Pb, and Cr in all the sites are due to unregulated illegal mining activities occurring in and around the study area. It is hoped that this study will prompt the basin management board to improve their management strategies in controlling unregulated illegal mining in the basin sediments.

## Introduction

Accumulation of heavy metals in the sediments of rivers which are exposed to mining and industrial waste is a common phenomenon in most developing countries (Islam et al. 2015). Such river sediments have become sinks for heavy metals, just like wetlands. The sediments sometimes act as carriers and sources for the heavy metals in the environment (Haiyan et al. 2013). The study of heavy metals in river sediments is very important because sediments serve as habitat for many benthic organisms like the mudfish. Unfortunately most of the time, the rivers are monitored without paying any attention to the sediments which are in constant interaction with the river. Studies have shown that rivers have been severely contaminated with heavy metals due to historic and modern mining and industrial operations (Miller et al. 2004). Heavy metals in river sediments enter through different pathways, either from point or non-point sources (Shazili et al. 2006). Examples of point sources could be the discharges of industrial waste such as metal mine wastes through pipes or drains, into rivers. Non-point sources such as silt-laden runoff from excavated lands and leachate from landfills also contribute to the levels of heavy metals usually discharged into water resources. The fate of heavy metals in an aquatic environment is affected by processes such as precipitation, sorption, and dissolution (Abdel-Ghani and Elchaghaby 2007). These processes are also affected by factors such as pH, temperature, dissolve oxygen concentration, and the disturbance of the water (Atkinson et al. 2007; Simpson et al. 2004). At higher pH, heavy metals precipitate and get adsorbed onto sediment surfaces. Metals are also released more easily into the water at lower pH and higher temperatures. When the dissolved oxygen concentration is low, i.e., less than 7 mg/L, heavy metals especially those bound to organic matter sediments are released into the overlying water and vice versa (Haiyan et al. 2013). A study by Atkinson et al. (2007) shows that physical disturbance of water releases metals more rapidly into water than biological disturbance. The study of heavy metals in sediments can serve as a guide in predicting the extent of pollution of the overlying water under different environmental conditions.

The present study assesses the heavy metal pollution level in the main Pra River and two of its tributaries in the Pra Basin of Ghana. The study area is the largest among the three southwestern river systems in Ghana and occupies an area of 23,000 km^2^ which is about 9.64% of the area of Ghana. Sediment pollution by heavy metals in the study area is now graduating into a major problem with the increased illegal mining activities in and around the rivers in the basin which are increasing the turbidity and the heavy metal levels, making the rivers physically unstable and chemically and biologically toxic. The present state of the rivers poses serious problems to the environment and the health of those villages which still depend on the rivers for cooking and bathing during water crises. To date, no detailed scientific analysis of the river sediments has been conducted. The aim of this study is to assess the concentrations of lead (Pb), cadmium (Cd), arsenic (As), chromium (Cr), iron (Fe), manganese (Mn), zinc (Zn), and nickel (Ni) using the enrichment factor (EF), pollution load index (PLI), and the geo-accumulation index (Igeo). Geo-accumulation index determines the metal levels of contamination or accumulation with reference to background levels of the same element in the environment. EF which is also an indication of enrichment of a selected metal with reference to a background metal such as iron complements the Igeo by indicating the source of enrichment as either natural or anthropogenic. The pollution load index assesses the cumulative pollution effect of the metals at each site by making reference to the EF of all the metals measured at each site.

## Materials and Methods

**Fig. 1:**
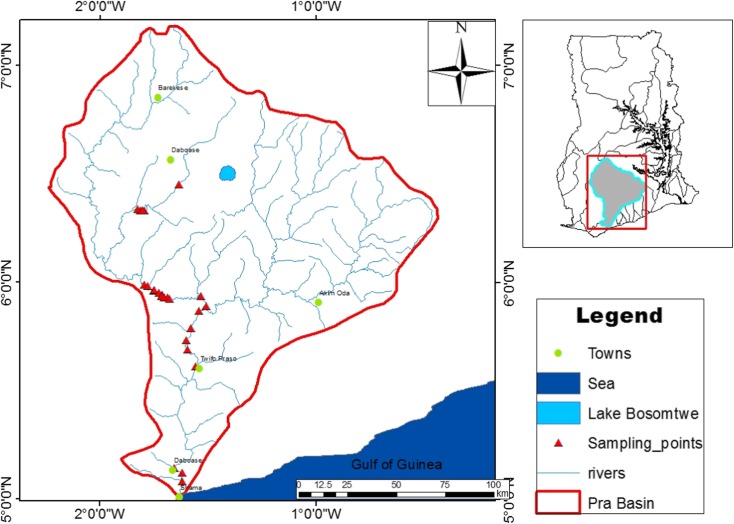
Map of Pra Basin

This study was conducted in the Pra Basin of Ghana. The hydrogeology of the Pra Basin is dominated by aquifers of the crystalline basement rocks and the Birimian Province. Sediment texture from the sampling site spans from sand, sandy loam, loamy sand, silty clay loam, and sandy clay loam. The Basin is located between latitudes 5° N and 7° 30′ N, and longitudes 2° 30′ W, and 0° 30′ W, in south-central Ghana. It is the largest among the three southwestern basins in Ghana (Ankobra, Tano, and Pra) and covers an area of 238,540 km^2^. The basin enjoys sub-equatorial wet climate with two raining seasons (May–June and September–November). The relative humidity in the basin is around 70 to 80% throughout the year. The annual rainfall range is between 1300 and 1900 mm with an annual mean value of 1500 mm. The only natural lake in Ghana, Bosomtwe, which is a major tourist attraction is located in the basin. The land area is largely dominated by agriculture (60%) with the remaining 40% being covered by human settlement (10%) and forest (30%). Towns like Twifo Praso and Kade in the basin are known for their large palm plantations. Gold mining both regulated and unregulated is the most prominent and highly patronized job in the basin. Figure 1 presents the study area map. The sampling order of the sites and their names from upstream to downstream in Fig. 1 are presented in Table 1. All sampling sites were either within or around an illegal mining site. A control site which has no such activities going on was also selected. From a total of 27 sampling points, 108 sediment samples were collected from January to April 2017 for the dry season and 108 from May to August 2017 for the wet season making a total of 216. The sediments were sampled from the riverbank by manual dredging using plastic scoop into polyethylene bags and air dried at room temperature and sieved through a 2-mm sieve for further analysis.

**Table 1:** Mean metal concentrations (mg kg^−1^) for dry season

Sites	Dry	Wet	Dry	Wet	Dry	Wet	Dry	Wet	Dry	Wet	Dry	Wet	Dry	Wet	Dry	Wet
	Ni	Ni	Cr	Cr	Mn	Mn	Fe	Fe	Cd	Cd	Pb	Pb	Zn	Zn	As	As
Lake(LAK)	*58.185*	*85.850*	*235.055*	*166.232*	44.378	**1**62.550	1665.793	1326.040	0.140	*7.945*	*111.903*	*147.370*	0.000	1.675	0.179	0.000
Oda (OD1)	*150.263*	*95.435*	*228.178*	*168.815*	214.255	14.993	1711.138	1704.055	*8.903*	*8.204*	*84.078*	*133.818*	*93.825*	31.210	0.241	0.003
Oda (OD2)	*91.678*	*89.113*	*241.080*	*186.880*	37.758	61.230	1557.973	1460.138	0.218	*8.775*	*776.205*	*148.205*	20.755	27.908	0.081	0.003
Oda (OD3)	*115.195*	*69.510*	*241.940*	*202.365*	96.435	111.345	1635.960	1319.765	*1.503*	*7.803*	*785.000*	*150.710*	34.423	11.020	0.189	0.001
Oda (OD4)	*85.255*	*68.680*	*192.903*	*185.162*	107.865	45.355	1272.795	1024.010	*3.048*	*8.923*	*81.390*	*157.183*	9.645	*89.363*	0.239	0.003
Praso Town (PT)	*54.628*	23.608	*223.875*	*135.265*	*3176.048*	*1714.375*	1508.510	1279.945	0.620	*4.928*	*384.665*	*111.875*	*89.865*	6.995	0.210	0.002
Praso Subinso (PS)	*91.778*	*52.060*	*251.403*	*150.747*	356.560	593.503	1643.730	1079.655	0.080	*5.270*	*164.205*	*113.965*	*229.058*	53.030	0.127	0.001
Twifo Agona (TAG)	*60.853*	20.740	*211.830*	*146.445*	61.085	61.378	1115.605	643.125	*3.495*	*5.613*	*64.458*	*115.633*	29.078	14.365	0.134	0.002
Twifo Kotokyire (TK)	*65.498*	*29.038*	*215.270*	*146.447*	80.315	145.343	1474.273	1282.910	*2.548*	*7.773*	*72.620*	*116.050*	23.463	32.245	0.074	0.002
Assin Awisam (TAW)	*51.563*	*104.918*	*215.273*	*174.84*	223.750	14.748	1126.935	935.755	*2.813*	*7.878*	*316.583*	*117.928*	15.348	49.095	0.319	0.002
Assin asaman (AAS)	*79.575*	*73.190*	*206.670*	*146.445*	84.373	160.345	1307.158	1263.715	*1.285*	*6.895*	*335.865*	*119.180*	23.040	19.943	0.272	0.002
Assin Nyardom (ANY)	*55.715*	*32.795*	*214.410*	*148.165*	49.415	10.303	1510.693	1135.858	0.500	*7.660*	*282.328*	*123.358*	52.908	5.755	0.140	0.003
Dunkwa Town (DT)	21.035	17.020	*254.845*	*183.437*	7.093	89.653	1252.325	1298.545	*3.498*	*7.040*	*415.688*	*127.743*	*89.730*	17.980	0.194	0.003
Dunkwa upstream (DU)	*53.540*	*53.838*	*242.800*	*186.022*	136.443	53.608	1434.985	734.408	*2.835*	*7.863*	*152.730*	*127.743*	*94.123*	11.600	0.181	0.001
Dunkwa Breman (DBR)	*87.235*	*150.365*	*244.523*	*178.275*	94.130	172.348	1147.093	1232.538	*2.348*	*6.865*	*394.550*	*133.173*	41.588	*118.385*	0.061	0.002
Dunkwa downstream (DDO)	*104.425*	*84.073*	*164.515*	*175.697*	91.923	20.995	1333.933	1345.260	*1.490*	*7.373*	*64.730*	*119.865*	*548.103*	25.603	0.125	0.003
Dunkwa Ankaase (DAN)	*52.353*	*123.000*	*276.353*	*206.667*	161.578	265.855	1673.068	1395.943	*2.050*	*7.305*	*377.615*	*135.470*	48.550	68.585	0.267	0.002
Dunkwa Kojokrom (DKO)	*76.663*	*58.878*	*243.660*	*176.557*	7.900	12.138	1480.423	1312.743	*2.755*	*8.188*	*368.655*	*138.810*	648.300	0.383	0.152	0.002
Appiah Nkwanta (ANK)	*73.500*	*126.950*	*260.005*	*206.667*	195.320	313.715	1413.263	1445.250	0.635	*8.193*	*406.140*	*138.393*	47.575	*95.018*	0.139	0.002
Dunkwa Edwuma (DED)	*85.058*	*77.450*	*243.660*	*196.345*	139.783	117.155	1578.255	1330.470	0.545	*8.348*	*381.775*	*139.225*	16.020	40.028	0.243	0.003
Dunkwa Akropong (DAK)	*107.983*	*97.213*	*222.153*	*173.975*	135.448	24.075	561.423	621.405	*2.803*	*7.428*	*258.885*	*141.105*	23.043	1.418	0.136	0.001
Dunkwa Kyekyere (DKY)	24.593	*103.765*	*229.035*	*178.277*	4.153	40.895	1482.793	511.523	0.893	*8.558*	*658.620*	*142.988*	48.428	60.513	0.304	0.002
Anhwia Nkwanta (AAN)	*71.333*	*84.468*	*252.973*	*174.837*	171.905	139.445	1613.560	1578.720	*7.083*	*4.905*	*713.723*	*145.890*	*108.503*	21.068	0.077	0.001
Beposo (BEP)	*155.668*	*72.118*	*125.923*	*141.282*	179.973	61.525	1085.095	815.428	*8.743*	*6.673*	*1234.093*	*374.675*	50.665	37.830	0.199	0.001
Daboase (DAB)	*39.908*	*53.740*	*144.535*	*136.122*	165.708	172.103	1353.083	1030.895	*7.815*	*5.973*	*34.870*	*68.185*	78.643	17.873	0.136	0.001
Atwereboanda (ATW)	*129.630*	*47.415*	*188.698*	*136.985*	28.228	48.820	730.488	600.035	*9.283*	*7.078*	*56.060*	*43.878*	*452.268*	11.493	0.244	0.001
Shama (SHA)	*114.938*	*66.683*	*134.130*	*116.335*	286.210	337.623	901.500	1032.758	*8.648*	*7.078*	*77.865*	*54.898*	*277.778*	*91.418*	0.071	0.000
AVG	*79.928*	*72.663*	*218.729*	*167.603*	234.742	183.904	1354.513	1138.552	*3.206*	*7.279*	*335.381*	*132.864*	*118.323*	35.622	0.175	0.002
STD	34.074	33.456	38.537	24.162	594.105	332.209	292.177	315.643	3.038	1.119	289.153		169.540	32.440	0.074	0.001
WHO	25	25	50	50	600	600	28,000	28,000	1.1	1.1	23	23	88	88	7	7

Italicized figures are above WHO standard

### Chemicals and Sample Digestion

Deionized water supplied by University of Cape Coast Technology Village was used in all the analyses. All standard solutions used were of the highest purity supplied by MES Equipment Limited, Ghana. The nitric and hydrochloric acids used for the digestion were all of the analytical grades and supplied by MES Equipment. The sieved sediment was further ground with mortar and pestle until fine particles (< 200 μm) were obtained (Ismaeel and Kusag 2015). About 2 g of the ground sediment was taken in a 100-mL beaker and 15 mL of concentrated HNO_3_ was added. The content was heated at 130 °C for 5 h until 2–3 mL remained in the beaker. The digested sediment was then passed through Whatman no. 41 filter paper and washed with a 0.1 M HNO_3_ solution and made to 100 mL volume using deionized water (Ali et al. 2016).

### Analytical Technique and Accuracy Check

The heavy metal determination was conducted using a dual atomizer and hydride generator atomic absorption spectrophotometer (model ASC-7000 No A309654, Shimadzu, Japan). All the samples were analyzed for arsenic (As), chromium (Cr), cadmium (Cd), lead (Pb), manganese (Mn), nickel (Ni), zinc (Zn), and iron (Fe). All reagents used were of the analytical grade from MES Equipment, Ghana. Ultrapure metal free deionized water was used for all analyses. All glass and plastic wares were cleaned by soaking them in warm 5% (*V*/*V*) aqueous nitric acid for 6–7 h and rinsed with ultrapure deionized water. The standard for the ASS calibration was prepared by diluting standard (1000 ppm) supplied by MES Equipment Limited, Ghana. All measured results were converted from milligram per liter and microgram per liter to milligram per kilogram. Matrix Spike recovery was in the range of 85–100%. The performance of the AAS was checked daily to ensure that the instrument is working according to the specifications.

### Assessment of Heavy Metal Pollution

The choice of background values plays important roles in geochemical data interpretation (Ali et al. 2016). The background value is the natural content of a substance in the soil which is completely dependent on the composition and mineralogical characteristics of the parent/source geological material (Maurizio 2016). The contribution of human activities to the levels of heavy metals in sediments and their pollution can be estimated using Igeo, EF, and PLI.

#### Geo-Accumulation Index

This index was first proposed for metal concentration determination in 2-μm fraction and later developed to the present form (Müller 1979). The method is used to determine the levels of contamination or accumulation of metals in soil. The formula is mathematically expressed as:

1 $$ Igeo=\mathit{\log}2\frac{\left[ Cn\right]}{1.5 Bn} $$

Where Cn is the measured concentration of metal n in the sediment, Bn is the geochemical background value of element n in the background sample (Yu et al. 2011), and 1.5 is the background matrix correction factor due to lithogenic effects. Müller (1979) gave seven classes for interpreting the geo-accumulation index which ranged as follows: Igeo ≤ 0, uncontaminated; 0 < Igeo < 1, uncontaminated to moderately contaminated; 1 < Igeo < 2, moderately contaminated; 2 < Igeo < 3, moderately to heavily contaminated; 3 < Igeo < 4, heavily contaminated; 4 < Igeo < 5, heavily to extremely contaminated; and Igeo ≥ 5, extremely contaminated.

#### Enrichment Factor and Pollution Load Index

The enrichment factor as proposed by Zoller (1974) is given by:

2 $$ EF=\frac{\left[ Ai\right]}{\left[ Ao\right]}/\frac{\left[ Bi\right]}{\left[ Bo\right]} $$

[Ai] and [Bi] are the concentrations of elements A and B at sampling station i; [Ao] and [Bo] are the background concentrations of elements A and B. Values estimated for EF from Eq. (1) provide the pollution state of the sediment. Values of 0.5 ≤ EF ≤ 1.5 are an indication that the metal concentration is a natural weathering process (Zhang and Liu 2002). A value above 1.5 indicates the influence of anthropogenic activity (Klerks and Levinton 1989; Taylor et al. 2010; Zhang and Liu 2002). There are five classes of contamination with reference to EF: EF < 2, depletion to minimal enrichment; EF = 2–5, moderate enrichment; EF = 5–20, significant enrichment; EF = 20–40, very high enrichment; EF > 40, extremely high enrichment. The pollution load index is defined as the *n*th root of the multiplication of the EF of metals involved

$$ \mathrm{PLI}={\left({\mathrm{EF}}_1\times {\mathrm{EF}}_2\times {\mathrm{EF}}_3\times {\mathrm{EF}}_4\times {\mathrm{EF}}_{\mathrm{n}}\right)}^{1/n} $$

According to Tomilson (1980), a PLI of 0 indicates excellence; a value of 1 indicates baseline levels of the concerned metals, whereas values above 1 are signs of progressive deterioration. Whereas EF gives the individual effects of the metals at a site, the PLI gives the overall effect of all metals studied at a site.

## Results and Discussion

The mean heavy metal concentrations for sediments in the study sites during the dry and wet seasons are presented in Table 1. Praso Town (PT) recorded the highest average metal concentration during the period under study. Dunkwa Akropong (DAK) and Atweneboanda (ATW) recorded the lowest metal concentrations during the dry and wet seasons respectively (Tables 1). The observed high metal concentrations in PT can be attributed to the uncontrolled and scattered illegal mining activities occurring in and around the area. The lowest metal concentration found in ATW river sediments may be due to dilution in the area as the town is the last point after which the river joins the sea. The river is a major source of water for domestic activities in ATW; the frequent visitation of the river banks and domestic activities such as washing and playing along the banks of the river as compared to other areas sampled may have contributed to the washing away of the top sediments and thereby reduce accumulation of metals. Generally, there is a significant difference in the dry season metal concentration (*M* = 293.12, SE = 18.31) and wet season metal concentration (*M* = 217.31, SE = 11.93); the difference in concentration in the dry season may be attributed to the intensification of illegal mining activities which occurred as a result of a government order to halt illegal mining after the dry season of 2017. Excessive washing of the surface soil during the wet season could also account for the lower concentrations in the wet season.

The iron (Fe) and arsenic (As) concentrations in the wet and dry seasons were lower than WHO standards. Regarding manganese (Mn), apart from site PT which recorded concentrations of about 5 and 3 times the background levels for both dry and wet seasons, all other sites recorded values or concentrations below the background levels. The high values of manganese recorded at PT may be due to the sloppy nature of the land which turns to experience high level of siltation from turbid water flowing from nearby illegal mining sites. Zinc (Zn) concentrations in sediments were above the background values for 9 out of 27 of the sites in the dry seasons and only 3 out of 27 of the sites in the wet season. In the case of nickel (Ni), only 2 sites recorded values below the background values (Table 1). Concerning chromium (Cr), all the sites recorded values above the background levels. Cr values as high as 5 and 4 times the background values were recorded for the dry and wet seasons (Table 1). Cadmium (Cd) recorded concentrations as high as 8 times the background values. Unlike the wet season, 8 out of the 27 sites in the dry season recorded Cd values below the background values. Lead (Pb) is the only metal whose concentration is above the background level for all the sites in both dry and wet seasons. The identified metals (Ni, Cr, Cd, Pb) are major components of the soil from which the gold is mined. Furthermore, the metal mercury, which is usually part of the soil sediment because of its use in the gold extraction, was absent. The absence of mercury in the soil is expected because miners now carry out the extraction of the gold far away from the mining location due to the threat posed by arm robbers. The most striking result to emerge from the data is the abnormally high value of Pb concentration at BEP during the dry season. The measured Pb concentration (Table 1) is about 54 times the background value. Metal concentration exceeding the background level is an indication that their presence in the sediments is due to human activities. The BEP environment is highly dominated by illegal mining activities. Exposure to high level of illegal mining activities especially through the use of sophisticated machines recorded the high metal concentrations or values (Table 1). The mean concentration of metals exceeding background level in the wet season is in the order Cr > Pb > Ni > Cd > Zn and in the dry season as Pb > Cr > Ni > Cd.

### Sediment Pollution Assessment

The calculated EF, PLI, and the background concentrations of metals in freshwater ecosystems are presented in Table 2. The EF ranged between 0 and 53.656 during the dry season and 0.003–45 during the wet season which indicates that the measured concentrations of four metals (Mn, Fe, Zn, and As) out of the eight in the studied area in both seasons were due to natural weathering process (0.5 ≤ EF ≤ 1.5), whereas the rest (Pb, Cd, Cr, and Ni) were due to anthropogenic activities (EF > 2). All the sites studied showed depletion to minimal enrichment for the metals Mn, Fe, Zn, and As for the dry and wet seasons. All sites showed moderate enrichment (EF = 2–5) for Cr in both dry and wet seasons. Five sites (TAG, TK, ANY, DT, and ATW) out of the 27 recorded depletion to minimal enrichment for Ni in the wet season with 21 out of the 27 sites recording moderate enrichment and only 1 site (PT) recording extremely high enrichment. Unlike the wet season, only 3 sites (DT, DKY, and DAB) out of the 27 recorded depletion to minimal Ni enrichment for the dry season, the remaining 24 sites recorded values within the range of moderate enrichment to significant enrichment (Table 2). However, there is no significant statistical difference in the dry season nickel enrichment (*M* = 3.19, SE = 0.26) and wet season nickel enrichment (*M* = 4.53, SE = 1.57) in the basin. In the case of Pb, there is a significant difference in the dry season enrichment (*M* = 14.58, SE = 2.41) and wet season enrichment (*M* = 5.77, SE = 0.66). Four out of the 27 sites recorded moderate Pb enrichment whereas 22 recorded significant enrichment with only 1 site Atweneboanda (ATW) recording depletion to minimum enrichment in the wet season. However, in the dry season, 8 sites recorded moderate Pb enrichment, 13 sites recorded significant enrichment, 4 recorded very high enrichment, and 1 recorded extremely high enrichment. In the dry season, Cd recorded depletion to minimal enrichment in 12 sites, recorded moderate enrichment in 6 sites, and recorded significant enrichment in 9 sites. However, it recorded moderate to significant enrichment for all the sites in the wet season (Table 2). The seasonal influence on Cd enrichment in the sediment is very significant: dry season Cd enrichment (*M* = 2.76, SE = 0.53) and wet season enrichment (*M* = 6.61, SE = 0.19) (Table 2). Irrespective of the high enrichment factors recorded for some sites, BEP was the only site polluted (PLI > 1) (Table 2) in both seasons. LAK which is upstream and served as the control site is the only sampling point which recorded excellent value for pollution (PLI = 0) in the dry season (Table 2). Though LAK did not record 0 in the wet season, the value of 0.374 was still within the baseline level. The 0.374 is expected because in the wet season, the lake receives a lot of runoff with high silt content from the surrounding mountains without any means of exiting such inflows. On the contrary, the calculated PLI for the remaining 26 sites though within the baseline level is due to unregulated illegal mining in the area.

**Table 2:** Enrichment factor (EF) and pollution load index (PLI) for dry and wet season

Sites	Enrichment factor (EF) and pollution load index (PLI) for dry and wet seasons																	
	Dry	Wet	Dry	Wet	Dry	Wet	Dry	Wet	Dry	Wet	Dry	Wet	Dry	Wet	Dry	Wet	Dry	Wet
	Ni	Ni	Cr	Cr	Mn	Mn	Fe	Fe	Cd	Cd	Pb	Pb	Zn	Zn	As	As	PLI	PLI
LAK	*2.327*	*3.434*	*4.609*	*3.325*	0.074	0.271	0.059	0.047	0.127	*7.223*	*4.865*	*6.407*	0.000	0.019	0.026	0.003	0.000	0.374
OD1	*6.011*	*3.817*	*4.474*	*3.376*	0.357	0.025	0.061	0.061	*8.093*	*7.458*	*3.656*	*5.818*	1.066	0.355	0.034	0.044	0.944	0.583
OD2	*3.667*	*3.565*	*4.727*	*3.738*	0.063	0.102	0.056	0.052	0.198	*7.977*	*33.748*	*6.444*	0.236	0.317	0.012	0.042	0.429	0.685
OD3	*4.608*	*2.78*	*4.744*	*4.047*	0.161	0.186	0.058	0.047	1.366	*7.093*	*34.130*	*6.553*	0.391	0.125	0.027	0.02	0.750	0.572
OD4	*3.410*	*2.747*	*3.782*	*3.703*	0.180	0.076	0.045	0.037	*2.770*	*8.111*	*3.539*	*6.834*	0.110	1.015	0.034	0.041	0.499	0.712
PT	*2.185*	*45*	*4.390*	*2.705*	5.293	2.857	0.054	0.046	0.564	*4.48*	*16.725*	*4.864*	1.021	0.079	0.030	0.028	0.971	0.968
PS	*3.671*	*2.082*	*4.929*	*3.015*	0.594	0.989	0.059	0.039	0.073	*4.791*	*7.139*	*4.955*	2.603	0.603	0.018	0.014	0.594	0.685
TAG	*2.434*	0.83	*4.154*	*2.929*	0.102	0.102	0.040	0.023	*3.177*	*5.102*	*2.803*	*5.028*	0.330	0.163	0.019	0.024	0.468	0.393
TK	*2.620*	1.162	*4.221*	*2.929*	0.134	0.242	0.053	0.046	*2.316*	*7.066*	*3.157*	*5.046*	0.267	0.366	0.011	0.027	0.450	0.583
TAW	*2.063*	*4.197*	*4.221*	*3.497*	0.373	0.025	0.040	0.033	*2.557*	*7.161*	*13.764*	*5.127*	0.174	0.558	0.046	0.023	0.661	0.524
AAS	*3.183*	*2.928*	*4.052*	*2.929*	0.141	0.267	0.047	0.045	1.168	*6.268*	*14.603*	*5.182*	0.262	0.227	0.039	0.027	0.591	0.615
ANY	*2.229*	1.312	*4.204*	*2.963*	0.082	0.017	0.054	0.041	0.455	*6.964*	*12.275*	*5.363*	0.601	0.065	0.020	0.036	0.479	0.352
DT	0.841	0.681	*4.997*	*3.669*	0.012	0.149	0.045	0.046	*3.180*	*6.4*	*18.073*	*5.554*	1.020	0.204	0.028	0.036	0.497	0.509
DU	*2.142*	*2.154*	*4.761*	*3.72*	0.227	0.089	0.051	0.026	*2.577*	*7.148*	*6.640*	*5.554*	1.070	0.132	0.026	0.02	0.698	0.458
DBR	*3.489*	*6.015*	*4.795*	*3.566*	0.157	0.287	0.041	0.044	*2.134*	*6.241*	*17.154*	*5.79*	0.473	1.345	0.009	0.023	0.600	0.861
DDO	*4.177*	*3.363*	*3.226*	*3.514*	0.153	0.035	0.048	0.048	1.355	*6.702*	*2.814*	*5.212*	6.228	0.291	0.018	0.042	0.673	0.551
DAN	*2.094*	*4.92*	*5.419*	*4.133*	0.269	0.443	0.060	0.05	1.864	*6.641*	*16.418*	*5.89*	0.552	0.779	0.038	0.032	0.765	0.902
DKO	*3.067*	*2.355*	*4.778*	*3.531*	0.013	0.02	0.053	0.047	*2.505*	*7.443*	*16.028*	*6.035*	7.367	0.004	0.022	0.031	0.712	0.285
ANK	*2.940*	*5.078*	*5.098*	*4.133*	0.326	0.523	0.050	0.052	0.577	*7.448*	*17.658*	*6.017*	0.541	1.08	0.020	0.023	0.636	0.945
DED	*3.402*	*3.098*	*4.778*	*3.927*	0.233	0.195	0.056	0.048	0.495	*7.589*	*16.599*	*6.053*	0.182	0.455	0.035	0.037	0.570	0.738
DAK	*4.319*	*3.889*	*4.356*	*3.48*	0.226	0.04	0.020	0.022	*2.548*	*6.752*	*11.256*	*6.135*	0.262	0.016	0.019	0.021	0.576	0.337
DKY	0.984	*4.151*	*4.491*	*3.566*	0.007	0.068	0.053	0.018	0.811	*7.78*	*28.636*	*6.217*	0.550	0.688	0.043	0.024	0.416	0.589
AAN	*2.853*	*3.379*	*4.960*	*3.497*	0.287	0.232	0.058	0.056	*6.439*	*4.459*	*31.031*	*6.343*	1.233	0.239	0.011	0.02	0.945	0.616
BEP	*6.227*	*2.885*	*2.469*	*2.826*	0.300	0.103	0.039	0.029	*7.948*	*6.066*	*53.656*	*16.29*	0.576	0.43	0.028	0.016	*1.027*	0.599
DAB	1.596	*2.15*	*2.834*	*2.722*	0.276	0.287	0.048	0.037	*7.105*	*5.43*	1.516	*2.965*	0.894	0.203	0.019	0.016	0.569	0.489
ATW	*5.185*	1.897	*3.700*	*2.74*	0.047	0.081	0.026	0.021	*8.439*	*6.434*	*2.437*	1.908	5.139	0.131	0.035	0.013	0.737	0.341
SHA	*4.598*	*2.667*	*2.630*	*2.327*	0.477	0.563	0.032	0.037	*7.861*	*6.434*	*3.385*	*2.387*	3.157	1.039	0.010	0.007	0.792	0.589

Italicized figures are above background values

The calculated geo-accumulation indexes for the four (Pb, Cd, Cr, and Ni) enriching metals during the two seasons are presented in Table 3. In either the dry or wet season, all the non-enriching metals (Mn, Fe, Zn, and As) did not contaminate (Igeo < 0) any of the sites studied except Zn which recorded a value of moderate contamination (1 < Igeo < 2) at a site during the dry season. The result of the geo-accumulation index calculation for both seasons (Table 3) shows that Cr and Cd values for all the 27 sites were within the uncontaminated to the moderately contamination class (0 ≥ Igeo < 2). Only 1 out of the 27 sites was moderately to heavily contaminated (Igeo < 3) with Pb in the wet season whereas the rest recorded values within the uncontaminated to moderately contaminated range (0 < Igeo < 2). Out of the 27 sites, only 2 were moderately to heavily contaminated with Ni, whereas the rest (25) were uncontaminated to moderately contaminated in the wet season (Table 3). The result (Table 3) shows site DAB as a drinking water intake point recording the highest contamination for Ni (11.140) and Pb (64.977) in the dry season. These high values could be attributed to the low flow rate at the time which aided the precipitation of these two metals.

**Table 3:** Dry and wet season geo-accumulation index (Igeo)

Sites	Dry	Wet	Dry	Wet	Dry	Wet	Dry	Wet	Dry	Wet	Dry	Wet	Dry	Wet	Dry	Wet
	Ni	Ni	Cr	Cr	Mn	Mn	Fe	Fe	Cd	Cd	Pb	Pb	Zn	Zn	As	As
LAK	1.578	0.837	0.607	0.871	− 0.23	− 0.405	− 0.215	− 0.201	− 0.281	0.441	0.589	0.477	0	− 0.159	− 0.17	− 0.113
OD1	0.499	0.742	0.623	0.854	− 0.483	− 0.169	− 0.217	− 0.216	0.411	0.432	0.778	0.511	− 2.03048	− 0.481	− 0.184	− 0.197
OD2	0.775	0.801	0.594	0.759	− 0.219	− 0.258	− 0.21	− 0.206	− 0.342	0.415	0.223	0.476	− 0.37467	− 0.446	− 0.142	− 0.193
OD3	0.618	1.123	0.592	0.698	− 0.31	− 0.332	− 0.214	− 0.2	− 7.402	0.446	0.222	0.47	− 0.5157	− 0.279	− 0.173	− 0.16
OD4	0.844	1.145	0.734	0.767	− 0.327	− 0.232	− 0.198	− 0.187	1.13	0.411	0.808	0.457	− 0.26493	− 1.777	− 0.183	− 0.192
PT	1.843	− 1.498	0.634	1.175	0.55	1.076	− 0.208	− 0.199	− 0.708	0.634	0.287	0.589	− 1.80275	− 0.236	− 0.177	− 0.174
PS	0.774	*2.113*	0.573	0.993	− 0.749	− 1.665	− 0.214	− 0.189	− 0.229	0.597	0.444	0.58	1.25759	− 0.76	− 0.157	− 0.149
TAG	1.432	− 1.17	0.668	1.036	− 0.258	− 0.258	− 0.191	− 0.166	0.924	0.566	1.109	0.573	− 0.45818	− 0.313	− 0.159	− 0.168
TK	1.243	− 2.71	0.657	1.036	− 0.287	− 0.38	− 0.207	− 0.199	1.596	0.447	0.931	0.571	− 0.40127	− 0.492	− 0.14	− 0.172
TAW	*2.177*	0.674	0.657	0.819	− 0.498	− 0.169	− 0.192	− 0.182	1.3	0.443	0.313	0.564	− 0.32212	− 0.701	− 0.198	− 0.166
AAS	0.921	1.037	0.684	1.036	− 0.293	− 0.402	− 0.2	− 0.198	− 2.772	0.485	0.305	0.559	− 0.39709	− 0.367	− 0.19	− 0.172
ANY	1.751	− 5.17	0.66	1.018	− 0.239	− 0.155	− 0.208	− 0.192	− 0.581	0.451	0.33	0.544	− 0.75815	− 0.221	− 0.161	− 0.186
DT	− 1.199	− 0.877	0.567	0.775	− 0.143	− 0.301	− 0.197	− 0.199	0.923	0.478	0.278	0.53	− 1.79573	− 0.348	− 0.174	− 0.186
DU	1.947	1.917	0.59	0.763	− 0.367	− 0.246	− 0.205	− 0.171	1.281	0.444	0.466	0.53	− 2.04949	− 0.285	− 0.171	− 0.16
DBR	0.821	0.499	0.587	0.801	− 0.307	− 0.419	− 0.193	− 0.196	1.966	0.486	0.284	0.513	− 0.60013	− 6.367	− 0.135	− 0.167
DDO	0.677	0.859	0.882	0.814	− 0.304	− 0.184	− 0.201	− 0.201	− 6.796	0.463	1.102	0.557	0.486877	− 0.423	− 0.156	− 0.194
DAN	*2.077*	0.584	0.531	0.684	− 0.404	− 0.568	− 0.215	− 0.204	3.193	0.466	0.29	0.507	− 0.693	− 1.059	− 0.189	− 0.18
DKO	0.969	1.537	0.588	0.81	− 0.146	− 0.161	− 0.207	− 0.2	1.352	0.433	0.293	0.498	0.435517	− 0.119	− 0.164	− 0.179
ANK	1.03	0.568	0.558	0.684	− 0.454	− 0.658	− 0.204	− 0.206	− 0.726	0.433	0.281	0.499	− 0.67923	− 2.108	− 0.16	− 0.165
DED	0.846	0.956	0.588	0.72	− 0.372	− 0.34	− 0.211	− 0.201	− 0.626	0.428	0.288	0.497	− 0.32867	− 0.581	− 0.184	− 0.188
DAK	0.655	0.728	0.638	0.824	− 0.366	− 0.191	− 0.161	− 0.165	1.308	0.461	0.344	0.492	− 0.39711	− 0.153	− 0.16	− 0.163
DKY	− 1.643	0.681	0.621	0.801	− 0.129	− 0.224	− 0.207	− 0.157	− 1.128	0.421	0.235	0.488	− 0.69126	− 0.889	− 0.196	− 0.168
AAN	1.078	0.854	0.57	0.819	− 0.419	− 0.372	− 0.213	− 0.211	0.476	0.636	0.229	0.481	− 3.53595	− 0.378	− 0.141	− 0.161
BEP	0.487	1.06	1.338	1.095	− 0.431	− 0.258	− 0.19	− 0.176	0.416	0.496	0.194	0.291	− 0.72386	− 0.555	− 0.175	− 0.153
DAB	*11.14*	1.926	1.057	1.163	− 0.41	− 0.419	− 0.202	− 0.187	0.446	0.539	*64.977*	1.017	− 1.33841	− 0.347	− 0.159	− 0.152
ATW	0.559	*2.955*	0.751	1.151	− 0.2	− 0.238	− 0.171	− 0.163	0.401	0.476	1.428	*2.883*	0.562861	− 0.284	− 0.184	− 0.146
SHA	0.619	1.204	1.192	1.579	− 0.605	− 0.707	− 0.18	− 0.187	0.418	0.476	0.852	1.492	0.931626	− 1.887	− 0.139	− 0.13

Italicized figures are above background values

The reason accounting for the difference in contamination across the seasons may be due to the following: (1) the washing away of the top sediments through the heavy downpour and high runoff in the wet season; (2) the low flow rate during the dry season which aids the process of precipitation and accumulation. The results of the geo-accumulation index shows the need for regular monitoring of the metals Ni and Pb and the illegal mining activities especially during the dry season at the sampling site DAB to avoid further accumulation, contamination, and subsequent pollution of such metals at the intake.

## Conclusions

The river sediment in the Pra Basin is enriched and contaminated with Ni, Cr, Cd, and Pb, which is an indication of the human activities in the basin. Generally, the mean concentrations of the metals were higher in the dry season than the wet season due to the low flow rate during the dry season which aids the process of precipitation and accumulation. It was only Beposo (BEP) which was found to be polluted (PLI < 1). Extreme contamination (Ni and Pb) occurred at Daboase (DAB) which serves as an intake for the water treatment. This is due to the high illegal mining activities occurring in and around DAB and its environs. The result (Table 3) of the study shows the need for general monitoring of illegal mining activities as well as all four metals (Ni, Cr, Cd, and Pb) especially Ni and Pb at DAB. The monitoring will not only address the problem of further accumulation and pollution of these metals but it will also solve public health concerns which arise from the intake of these metals which are carcinogenic. Crop production on these soils is a potential route for these metals to enter the ecosystem, hence the need for monitoring of activities in and around the river sediments, especially during the dry seasons. Finally, monitoring is required to reduce high-level siltation in the river basins which could lead to the drying of such rivers; a situation which threatens some rivers in some parts of Ghana at the moment.
